# An Industrial Fault Diagnostic System Based on a Cubic Dynamic Uncertain Causality Graph

**DOI:** 10.3390/s22114118

**Published:** 2022-05-28

**Authors:** Xusong Bu, Hao Nie, Zhan Zhang, Qin Zhang

**Affiliations:** 1 Department of Computer Science and Technology, Tsinghua University, Beijing 100084, China; buxusong@mail.tsinghua.edu.cn; 2 Institute of Nuclear and New Energy Technology, Tsinghua University, Beijing 100084, China; nh17@mails.tsinghua.edu.cn (H.N.); zhangzhan190@tsinghua.edu.cn (Z.Z.)

**Keywords:** industrial fault diagnosis, cubic DUCG, causal inference, expert knowledge

## Abstract

This study presents an industrial fault diagnosis system based on the cubic dynamic uncertain causality graph (cubic DUCG) used to model and diagnose industrial systems without sufficient data for model training. The system is developed based on cloud native technology. It contains two main parts, the diagnostic knowledge base and the inference method. The knowledge base was built by domain experts modularly based on professional knowledge. It represented the causality between events in the target industrial system in a visual and graphical form. During the inference, the cubic DUCG algorithm could dynamically generate the cubic causal graph according to the real-time data and perform the logic and probability calculations based on the generated cubic DUCG models, visually displaying the dynamic causal evolution of faults. To verify the system’s feasibility, we rebuild a fault-diagnosis model of the secondary circuit system of No. 1 at the Ningde nuclear power plant based on the new system. Twenty-four fault cases were used to test the diagnostic accuracy of the system, and all faults were correctly diagnosed. The results showed that it was feasible to use the cubic DUCG platform for fault diagnosis.

## 1. Introduction

With the increasing complexity of industrial systems, the systems’ safety has attracted extensive attention. Suppose some minor faults in the system cannot be detected and eliminated in time. In that case, it may cause the failure and paralysis of the entire system, and even lead to substantial disastrous consequences [1,2,3,4]. Improving the safety and reliability of the system and preventing and eliminating the occurrence and development of faults that affect the regular operation of the system has become a crucial problem to be solved. Fault monitoring and diagnostic technology is an effective method to improve the safety and reliability of complex systems [5,6,7]. Early fault-diagnosis expert systems were mostly rule-based and case-based [8,9,10]. The systems’ diagnostic rules and cases were constructed by experts based on experience. The advantage is that they are less dependent on data and are interpretable. When the industrial system is relatively simple, it is practical to use those methods. When the industrial system is complex, those methods are prone to knowledge conflict, repetition, and circulation [11]. In addition, the increase in knowledge reduces the reasoning efficiency of the system [12,13]. With the development of machine learning, some machine-learning algorithms are applied in fault-diagnosis systems, including SVM [14,15,16], ANN [17,18], and DNN [19,20,21]. Those methods use machine-learning theory to adaptively learn the diagnostic knowledge from the collected data, rather than using the experience and knowledge of engineers. The fault diagnostic system based on machine learning has high diagnostic efficiency. When the training data are sufficient, the diagnostic accuracy of the model is high. However, the models constructed based on these methods are black-box models, which lack interpretability of the diagnostic results [22].

In some customized complex systems, such as nuclear power plants or spacecraft [23], these systems are usually unique. They have high complexity, safety, and reliability, and almost no failures. So, the data used to train the diagnostic model are insufficient [24]. The fault diagnosis of such systems requires high reliability and the entire fault diagnostic process to be interpretable, helping the operator understand the fault transmission process to take measures to eliminate the fault quickly. Obviously, in the fault diagnosis of such systems, the fault diagnostic method based on machine learning is not feasible. However, the construction of such systems condenses a large amount of expert knowledge, experience, and statistical data. Therefore, using domain expert knowledge, experience, and statistical data to construct a diagnostic model intuitively and realize an efficient and reliable diagnosis is a feasible method to solve such a diagnostic system [25].

The cubic DUCG is a probabilistic graphical model [26] developed from the dynamic uncertain causality graph theory [27]. It can intuitively describe the uncertain causality of events using probabilities and different graphical symbols, and has a solid ability to express the propagation of causal uncertainty, as well as the advantages of visualization, interpretability, and high computational efficiency. It has a complete mathematical foundation and theoretical system, and provides a concise expression and reasoning method of uncertain knowledge in the form of causal graphs. It can realize the causal inference problems of discrete, continuous, and fuzzy variables. At the same time, it can handle causal loops, allowing directed loops in the model [28,29,30]. These features enable the cubic DUCG to implement accurate inference of multiconnected causality efficiently. The cubic DUCG’s reasoning knowledge base can be constructed by domain experts based on their knowledge, experience, and statistical data [31,32]. Therefore, it is suitable for scenarios in which causal inference is required but there are insufficient data to train the diagnostic model.

In this study, we built a time-series-based industrial fault diagnosis platform based on cubic DUCG theory. The following sections of this paper are arranged as follows: Section 2 introduces the cubic DUCG theory and describes the reasoning algorithm used in this diagnosis system; Section 3 presents the design and implementation of the diagnosis system; Section 4 provides the system verification; and Section 5 summarizes the study and suggests directions for future research.

## 2. The Cubic DUCG Modeling and Fault-Diagnosis Method

### 2.1. Cubic DUCG Modeling

**Figure 1:**
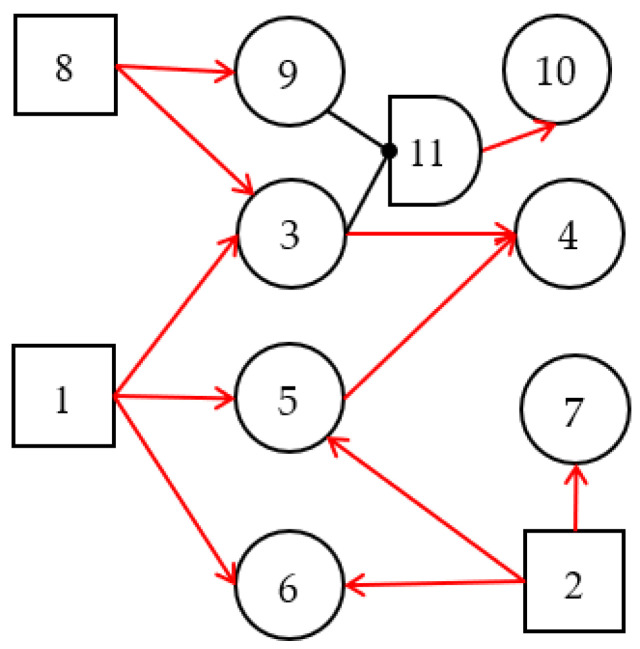
An example of the DUCG model. It describes the fault-propagation process through graphical symbols.

The cubic DUCG theory was developed from DUCG theory. It used the DUCG model as the inference knowledge base. The DUCG model represents the causal relationship between events, and can be built graphically and visually by domain experts using their knowledge, experience, and statistical data. Figure 1 is an example of the DUCG; its parameters are shown in Appendix A. As shown in Figure 1, in the DUCG model, different variables are represented by different shapes with numbers as indexes. Various variables give the DUCG a complete causal expression ability and make the causal expression more accurate. When constructing a DUCG model, domain experts only need to determine the events to be expressed and the causal relationship between events. Then, the appropriate variables and relationships to represent them are selected according to the construction rules of the DUCG model. The variables and their physical meanings are shown in Table 1.

**Figure 2:**
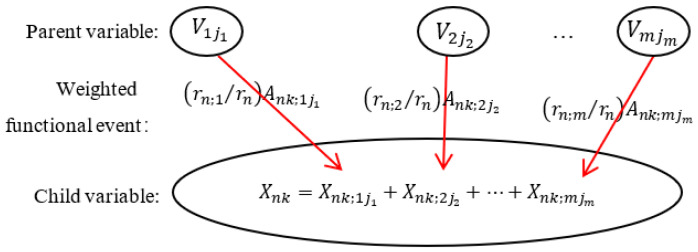
The causal mechanism between variables of the DUCG, where *X_nk_* is the child variable and *Vij_i_* is the parent variable.

The causal mechanism between variables of the DUCG is shown in Figure 2. The child event *X_nk_* may be caused by one or more of its parent events *V_ij_* (*V∈*{*B*, *X*, *D*, *G*}-type variable). The parent events cause the child event through the weighted functional events *F_nk_*_;*ij*_. *F_nk_*_;*ij*_ = (*r_n_*_;*i*_/*r_n_*) *A_nk_*_;*ij*_, where *A_nk;ij_* is the virtual random functional event representing the causal mechanism that *V_ij_* independently causes *X_nk_*. *a_nk;ij_* = *Pr* {*A_nk;ij_*} is the probability that *V_ij_* causes *X_nk_* to occur independently. The virtual random functional events between the parent *V_i_* and the child *X_n_* are represented by the matrix $A_{n;i}=\left(\begin{array}{ccc} A_{n0;i0} & \cdots & A_{n0;iJ}\\ \vdots & A_{nk;ij} & \vdots \\ A_{nK;i0} & \cdots & A_{nK;iJ} \end{array}\right)$. The matrix allows incomplete expression; if there is no causality between *V_ij_* and *X_nk_*, then *A_nk_*_;*ij*_ does not exist, and *A_nk_*_;*ij*_ is replaced with the symbol “-” in the matrix. When constructing causalities, we only need to give the parameters of the states that we care about to reduce the difficulty of knowledge-base construction. *r_n_*_;*i*_/*r_n_* ($r_{n}=\sum_{i}r_{n;i}$) is the weight parameter used to normalize the effect of parent variables on child variables. The relationship between parents is the logical weighted exclusive *OR* of the DUCG weighted set theory [33]. The weighted functional event and the logical weighted exclusive *OR* enable the DUCG to freely modify (add, delete, or update) the influence of parent variables on child variables.

In order to infer to which root causes the child event is related, the child event performs logic expression expansion along the opposite direction of the causal chain. After expression expansion, the child event is expressed by its parent events, and the expansion process can be executed recursively until the parent events are the *B*-type variables. The *B*-type variables are the root causes of other variables and the objects of inference calculation. The expansion expression is shown in Equation (1) [27]:

(1) $$X_{nk}=\sum_{i}\sum_{j_{i}}X_{nk;ij_{i}}=\sum_{i}\sum_{j_{i}}F_{nk;ij_{i}}V_{ij_{i}}=\sum_{i}\sum_{j_{i}}\left(r_{n;i}/r_{n}\right)A_{nk;ij_{i}}V_{ij_{i}}$$

For simplicity, Equation (1) can be briefly written as Equation (2):

(2) $$X_{nk}=\sum_{i}\sum_{j}F_{nk;ij}V_{ij}=\sum_{i}\sum_{j}\left(r_{n;i}/r_{n}\right)A_{nk;ij}V_{ij}$$

Logic expression expansion is an essential step of cubic DUCG reasoning. It can recursively expand the observation evidence *E* (*E* = *E*′*E″*, *E*′ = {X*_ij_*, j ≠ 0} is the collection of abnormal evidence, and *E″* = {*X_i_*_0_} is the collection of normal evidence) to the root faults. The result of the logic-expanded expression is used to calculate the conditional probability of each hypothesis under the current evidence.

### 2.2. The Inference Method of the Cubic DUCG

The reasoning process of the cubic DUCG reflects the temporal correlations among events, and is capable of representing the sequential causality interactions in fault-spreading processes. The reasoning step of the cubic DUCG is described as follows: (1) DUCG decomposition. The original DUCG is decomposed into several sub-DUCGs; each sub-DUCG contains one root event *B_i_*, remarked as *DUCG*(*B_i_*); (2) Obtain *Slice_DG* (*B_i_*, *t_m_*). The *Slice_DG* (*B_i_*, *t_m_*) is the intraslice causality graph at time *t_m_*. According to the evidence *E* (*t_m_*) at *t_m_*, the *DUCG* (*B_i_*)s are decomposed into several *Slice_DG* (*B_i_*,*t_m_*)s based on the cubic DUCG simplification rules [26,34]. Each *Slice_DG* (*B_i_*,*t_m_*) contains one root fault, and it describes the causality between the root fault *B_i_* and the evidence *E* (*t_m_*) at *t_m_*; (3) *Cubic_DG* (*B_i_*,*t_m_*) generation. The *Cubic_DG* (*t_m_*) is the generated cubic causality graph at *t_m_*. It is generated by merging the *Cubic_DG* (*B_i_*,*t_m_*_-1_) generated at *t_m_*_-1_ and *Slice_DG* (*B_i_*,*t_m_*) obtained at *t_m_*. *Cubic_DG* (*t_m_*) describes the propagation process of the evidence related to root fault *B_i_* from time t_1_ to time t_m_. In particular, when *t_m_*=t_1_, *Cubic_DG* (*B_i_*,*t*_1_) = *Slice_DG* (*B_i_*,*t*_1_); (4) Select a valid *Cubic_DG* (*B_i_*,*t_m_*)s. The reasoning of the cubic DUCG is based on the unary assumption that there can only be one root fault at the same time. Therefore, if the *Cubic_DG* (*B_i_*,*t_m_*) cannot explain all the evidence *E* (*t_m_*) at *t_m_*, it is regarded as an invalid *Cubic_DG* (*B_i_*,*t_m_*) and discarded. Only the *Cubic_DG* (*B_i_*,*t_m_*) that can explain all abnormal evidence *E* (*t_m_*) is regarded as a valid *Cubic_DG* (*B_i_*,*t_m_*); (5) Obtain the hypothesis space *S_H_* (*t_m_*). The root fault *B_i_* in a valid *Cubic_DG* (*B_i_*,*t_m_*) is regarded as the hypothesis *H_kj_*, and forms the hypothesis space, *S_H_* (*t_m_*) = {*H_kj_*}; (6) The logical expansion of expression. Logical expansion of *E* (*t_m_*) and *H_kj_E* (*t*_m_) are based on each respective *Cubic_DG* (*B_i_*,*t_m_*), until the expression contains only the form of the sum of variable products of {*B*-,*D*-,*A*-,*r*-}-type variables. Those expressions are used for the probability calculation; (7) Probability calculation. Calculate the conditional probability $\mathrm{Pr}\left\{H_{kj}\left(t_{m}\right)\right\}$ of each *H_kj_* in *S_H_* (*t_m_*) to evaluate which root fault is more likely to occur. The probability is calculated using Equation (3):

(3) $$\mathrm{Pr}\left\{H_{kj}\left(t_{m}\right)\right\}=h_{kj}^{s}\left(t_{m}\right)=\xi_{i}\left(B_{i},t_{m}\right)\frac{\mathrm{Pr}\left\{H_{kj}E\left(t_{m}\right)\right\}}{\mathrm{Pr}\left\{E\left(t_{m}\right)\right\}}$$

(4) $$\xi_{i}\left(B_{i},t_{m}\right)=\frac{\mathrm{Pr}\left\{E\left(t_{m}\right)\right\}}{\sum_{i}\mathrm{Pr}\left\{E\left(t_{m}\right)\right\}}=\frac{\varsigma \left(B_{i},t_{m}\right)}{\sum_{i}\varsigma \left(B_{i},t_{m}\right)}$$

In Equation (3), *E* (*t_m_*) is the abnormal evidence at time *t_m_*, and $E\left(t_{m}\right)=\prod_{i}X_{i,j_{i}}$. $\mathrm{Pr}\left\{E\left(t_{m}\right)\right\}=\varsigma \left(B_{i},t_{m}\right)$ is the joint probability of *E* (*t*_m_) on *Cubic_DG* (*B_i_*,*t_m_*). $\mathrm{Pr}\left\{H_{kj}E\left(t_{m}\right)\right\}$ is the joint probability of *H_kj_E* (*t*_m_) on *Cubic_DG* (*B_i_*,*t_m_*). $\xi_{i}\left(B_{i},t_{m}\right)$ is the weight factor of *E*(*t_m_*) on different *Cubic_DG* (*B_i_*,*t_m_*)s calculated using Equation (4). If there is only one *Cubic_DG* (*B_i_,t_m_*), then $\xi_{i}\left(B_{i},t_{m}\right)$ = 1. The sorted results are the reasoning result of the cubic DUCG at *t*_m_. Those *Cubic_DG* (*B_i_*,*t_m_*)s are used as the graphical explanations for inference results; (8) Repeat the procedures until the root fault is confirmed or no more abnormal evidence is received. The pseudo algorithm description of the calculation process is shown in Algorithm 1.
**Algorithm 1**: The Inference of the Cubic DUCG. (Note: The pseudo algorithm for inference process of the cubic DUCG.)**Input**: the original DUCG, the evidence $E\left(t_{m}\right)=\prod_{i}X_{i,j_{i}}$ at time *t_m_*.
**Steps**:Decompose the original DUCG to get the list of *DUCG* (*B_i_*);**for** *DUCG* (*B_i_*) **in** list of *DUCG* (*B_i_*)   *Slice_DG* (*B_i_*, *t_m_*)←GenerateValidSlice_DG (*DUCG* (*B_i_*), *E* (*t*_m_));   **Cubic_DG (B_i_*, *t_m_*)←GenerateCubic_DG (*Slice_DG* (*B_i_*, *t_m_*), Cubic_DG (B_i_*, *t_m_*_-1_));   *S_H_* (*t_m_*)←GetHypothesis (*Cubic_DG* (*B_i_*, *t_m_*));**end****for** *Cubic_DG* (*B_i_, t_m_*) **in** list of *Cubic_DG* (*B_i_, t_m_*)Logical expand *E* (*t_m_*) and *H_kj_E* (*t*_m_) based on each *Cubic_DG* (*B_i_*,*t_m_*);**end****for** *H_kj_* (*t_m_*) **in** *S_H_* (*t_m_*)   $\xi_{i}\left(B_{i},t_{m}\right)=\frac{\mathrm{Pr}\left\{E\left(t_{m}\right)\right\}}{\sum_{i}\mathrm{Pr}\left\{E\left(t_{m}\right)\right\}}=\frac{\varsigma \left(B_{i},t_{m}\right)}{\sum_{i}\varsigma \left(B_{i},t_{m}\right)}$   $\mathrm{Pr}\left\{H_{kj}\left(t_{m}\right)\right\}=h_{kj}^{s}\left(t_{m}\right)=\xi_{i}\left(B_{i},t_{m}\right)\frac{\mathrm{Pr}\left\{H_{kj}E\left(t_{m}\right)\right\}}{\mathrm{Pr}\left\{E\left(t_{m}\right)\right\}}$**end**Sort the list of $h_{kj}^{s}\left(t_{m}\right)$ in descending order.
**Output:** The sorted list of $h_{kj}^{s}\left(t_{m}\right)$ and *Cubic_DG(B_i_*, *t_m_*) at *t*_m_.

An example is used to exemplify the dynamic inference process of the cubic DUCG in a continuous time series. The original DUCG knowledge base is shown in Figure 1. It contains three root faults: *B*_1_, *B*_2,_ and *B*_8_. Other variables include some intermediate processes or results caused by root faults. The inference process contains three moments: *t*_1_, *t*_2_, and *t*_3_.

At time *t*_1_, suppose the received evidence is *E* (*t*_1_) = *X*_3,0_*X*_5,1_*X*_6,0_.

**Figure 3:**
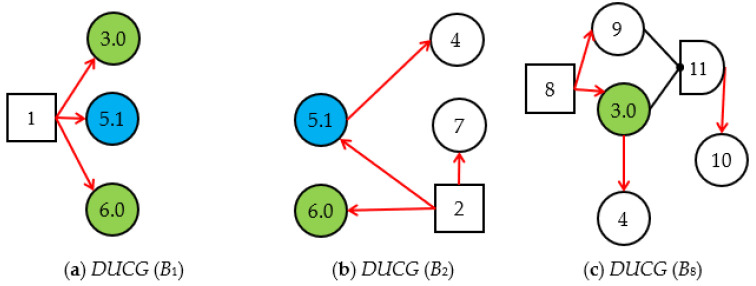
The original DUCG decomposed into three *DUCG (B_i_)* s.

Step 1.1. DUCG decomposition. By decomposing the original DUCG shown in Figure 1, we obtain three sub-DUCGs: *DUCG* (*B*_1_), *DUCG* (*B*_2_), and *DUCG* (*B*_8_). Each *DUCG* (*B_i_*) describes the relationships between the root fault *B_i_* and its related variables; the results are shown in Figure 3. Usually, the green circle stands for the normal evidence, the circle with other colors stands for the abnormal evidence, and the circle without colors stands for the state of the variable is known.

**Figure 4:**
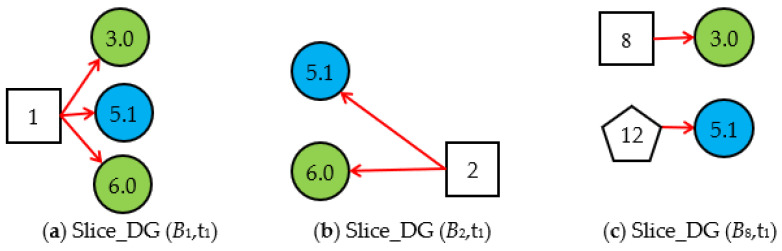
Three Slice_DG (*B_i_*,*t*_1_)s based on the evidence *E* (t_1_) = *X*_3,0_*X*_5,1_*X*_6,0_.

Step 1.2. Obtain the valid *Slice_DG (B_i_*,*t*_1_)s and generate *Cubic_DG (B_i_*,*t_1_*)s at *t*_1_. By simplifying *DUCG* (*B_i_*)s according to the simplification rules of the cubic DUCG, we obtain the three *Slice_DG* (*B_i_*,t_1_)s shown in Figure 4; The *Slice_DG* (*B_i_*,t_1_) shows the relationship between the root fault *B_i_* and the current evidence *E* (t_1_) at t_1_. The *Slice_DG* (*B*_1_,t_1_) and the *Slice_DG* (*B*_2_,t_1_) can explain the abnormal evidence, so they are the valid *Slice_DG* (*B_i_*,t_1_)s. In *Slice_DG* (*B*_8_,t_1_), the abnormal evidence *X*_5,1_ cannot be explained by the root fault *B*_8_, so it is the invalid *Slice_DG* (*B_i_*,t_1_) and is deleted.

**Figure 5:**
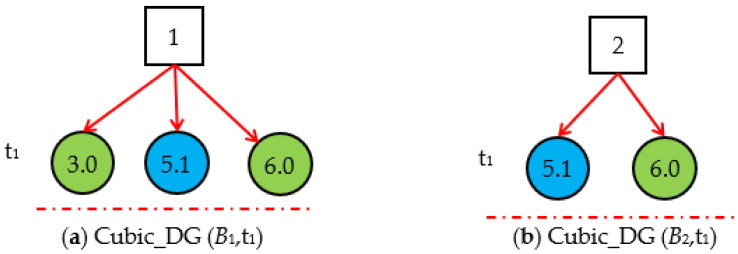
The Cubic_DG (*B_i_*,t_1_)s generated by the valid Slice_DG (*B_i_*,t_1_)s at t_1_.

Step 1.3. *Cubic_DB* (*B_i_*,t_1_) generation. At time t_1_, the valid *Slice_DG (B_i_*,t_1_)s are used as *Cubic_DG* (*B_i_*,*t*_1_)s, as shown in Figure 5. From the two *Cubic_DG(B_i_*,*t*_1_)s, we obtain the hypothesis spaces *S_H_*(*t_1_*)={*H*_1,1_, *H*_2,1_, *H*_2,1_}={ *B*_1,1_, *B*_2,1_, *B*_2,2_}.

Step 1.4. Logical expression expansion and probability calculation. The hypothesis *H*_1,1_ is included in *Cubic_DG* (*B*_1_,*t*_1_), and the hypotheses *H*_2,1_ and *H*_2,2_ are included in *Cubic_DG* (*B*_2_,*t*_1_). In order to calculate the Pr {*H*_1,1_ (*t*_1_)}, we should use expression expansion of *E* (*t*_1_) and *H*_1,1_*E* (*t*_1_) based on *Cubic_DG* (*B*_1_,*t*_1_), and then calculate Pr {*E*(*t*_1_)}, Pr {*H*_1,1_ *E* (*t*_1_)}. Equations (5) and (6) are the logic expansion of *E* (*t*_1_) and *H*_1,1_ *E* (*t*_1_) according to Equation (2). Equations (7) and (8) are the numerical calculation process of the expanded expressions; the parameters used in calculation can be seen in Appendix A.

(5) $$E\left(t_{1}\right)=X_{3,0}X_{5,1}X_{6,0}=F_{3,0;1,1}B_{1,1}F_{5,1;1,1}B_{1,1}F_{6,0;1,1}B_{1,1}=F_{3,0;1,1}F_{5,1;1,1}F_{6,0;1,1}B_{1,1}$$

(6) $$H_{1,1}E\left(t_{1}\right)=B_{1,1}X_{3,0}X_{5,1}X_{6,0}=F_{3,0;1,1}F_{5,1;1,1}F_{6,0;1,1}B_{1,1}$$

(7) $$\begin{array}{l} \varsigma \left(B_{1},t_{1}\right)=\mathrm{Pr}\left\{E\left(t_{1}\right)\right\}=\mathrm{Pr}\left\{X_{3,0}X_{5,1}X_{6,0}\right\}=\mathrm{Pr}\left\{F_{3,0;1,1}F_{5,1;1,1}F_{6,0;1,1}B_{1,1}\right\}\\ =f_{3,0;1,1}f_{5,1;1,1}f_{6,0;1,1}b_{1,1}\\ =\left(r_{1}/r_{3}\right)a_{3,0;1,1}\left(r_{1}/r_{5}\right)a_{5,1;1,1}\left(r_{1}/r_{6}\right)a_{6,0;1,1}b_{1,1}\\ \left(r_{1}/r_{3}\right)\times \left(1-a_{3,1;1,1}\right)\times \left(r_{1}/r_{5}\right)\times a_{5,1;1,1}\times \left(r_{1}/r_{6}\right)\times \left(1-a_{6,1;1,1}\right)\times b_{1,1}\\ =\left(1/1\right)\times \left(1-0.5\right)\times \left(1/1\right)\times 0.1\times \left(1/1\right)\times \left(1-0.4\right)\times 0.2\\ =0.006 \end{array}$$

(8) $$\mathrm{Pr}\left\{H_{1,1}E\left(t_{1}\right)\right\}=\mathrm{Pr}\left\{B_{1,1}X_{3,0}X_{5,1}X_{6,0}\right\}=\mathrm{Pr}\left\{F_{3,0;1,1}F_{5,1;1,1}F_{6,0;1,1}B_{1,1}\right\}=0.006$$

Similarly, for *H*_2,1_ and *H*_2,2_ in *Cubic_DG* (*B*_2_,*t*_1_), the results of expression expansion for *E* (*t*_1_), *H*_2,1_*E* (*t*_1_), and *H*_2,2_*E* (*t*_1_), as well as the results of Pr {*E* (*t*_1_)}, Pr {*H*_2,1_ *E* (*t*_1_)}, Pr {*H*_2,2_ *E* (*t*_1_)}, are shown in Equations (9)–(14).

(9) $$E\left(t_{1}\right)=X_{5,1}X_{6,0}=F_{5,1;2,1}F_{6,0;2,1}B_{2,1}+F_{5,1;2,2}F_{6,0;2,2}B_{2,2}$$

(10) $$H_{2,1}E\left(t_{1}\right)=B_{2,1}X_{5,1}X_{6,0}=F_{5,1;2,1}F_{6,0;2,1}B_{2,1}$$

(11) $$H_{2,2}E\left(t_{1}\right)=B_{2,2}X_{5,1}X_{6,0}=F_{5,1;2,2}F_{6,0;2,2}B_{2,2}$$

(12) $$\varsigma \left(B_{2},t_{1}\right)=\mathrm{Pr}\left\{E\left(t_{1}\right)\right\}=\mathrm{Pr}\left\{F_{5,1;2,1}F_{6,0;2,1}B_{2,1}+F_{5,1;2,2}F_{6,0;2,2}B_{2,2}\right\}=0.04$$

(13) $$\mathrm{Pr}\left\{H_{2,1}E\left(t_{1}\right)\right\}=\mathrm{Pr}\left\{F_{5,1;2,1}F_{6,0;2,1}B_{2,1}\right\}=0.025$$

(14) $$\mathrm{Pr}\left\{H_{2,2}E\left(t_{1}\right)\right\}=\mathrm{Pr}\left\{F_{5,1;2,2}F_{6,0;2,2}B_{2,2}\right\}=0.015$$

According to Equation (3), we obtain the posterior probability of *H*_1,1_, *H*_2,1_, and *H*_2,2_ shown in Equations (15)–(17):

(15) $$\mathrm{Pr}\left\{H_{1,1}\left(t_{1}\right)\right\}=h_{1,1}^{s}\left(t_{1}\right)=\xi_{1}\left(B_{1},t_{1}\right)\frac{\mathrm{Pr}\left\{H_{1,1}E\left(t_{1}\right)\right\}}{\mathrm{Pr}\left\{E\left(t_{1}\right)\right\}}=\frac{\varsigma \left(B_{1},t_{1}\right)}{\varsigma \left(B_{1},t_{1}\right)+\varsigma \left(B_{2},t_{1}\right)}\times \frac{\mathrm{Pr}\left\{H_{1,1}E\left(t_{1}\right)\right\}}{\mathrm{Pr}\left\{E\left(t_{1}\right)\right\}}=0.13$$

(16) $$\mathrm{Pr}\left\{H_{2,1}\left(t_{1}\right)\right\}=h_{2,1}^{s}\left(t_{1}\right)=\xi_{1}\left(B_{2},t_{1}\right)\frac{\mathrm{Pr}\left\{H_{2,1}E\left(t_{1}\right)\right\}}{\mathrm{Pr}\left\{E\left(t_{1}\right)\right\}}=0.543$$

(17) $$\mathrm{Pr}\left\{H_{2,2}\left(t_{1}\right)\right\}=h_{2,2}^{s}\left(t_{1}\right)=\xi_{1}\left(B_{2},t_{1}\right)\frac{\mathrm{Pr}\left\{H_{2,2}E\left(t_{1}\right)\right\}}{\mathrm{Pr}\left\{E\left(t_{1}\right)\right\}}=0.326$$

In the sorted results for $h_{2,1}^{s}\left(t_{1}\right)>h_{2,2}^{s}\left(t_{1}\right)>h_{1,1}^{s}\left(t_{1}\right)$, we can see that the hypotheses *H*_1,1_, *H*_2,1_, and *H*_2,2_ may cause the occurrence of the abnormal evidence *X*_5,1_ at time t_1_, and *H*_2,1_ is the most likely. The root fault *B*_8_ is excluded because it could not explain abnormal evidence.

At time *t*_2_, suppose the received abnormal evidence is *X*_6,1_, and combined with the evidence at t_1_, the total evidence at t_2_ is *E* (*t*_2_) = *X*_3,0_ *X*_5,1_ *X*_6,1_. The reasoning calculation process at t_2_ is as follows.

**Figure 6:**
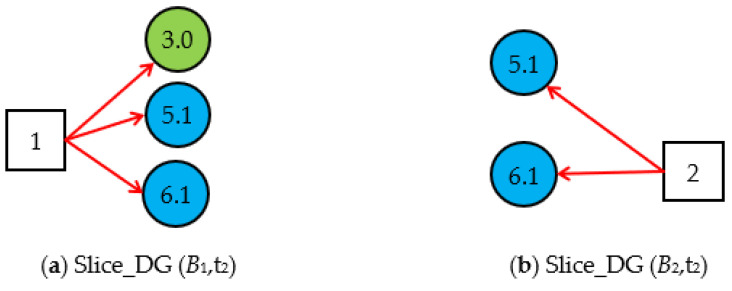
Slice_DG (*B_i_*,t_2_)s at t_2_.

Step 2.1. By simplifying *DUCG* (*B_i_*)s based on the evidence *E* (*t*_2_), we obtain the *Slice_DG* (*B*_1_,t_2_) and *Slice_DG* (*B*_2_,t_2_) shown in Figure 6.

**Figure 7:**
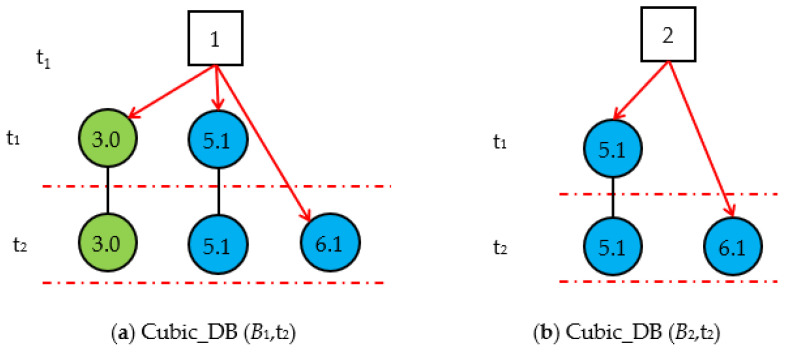
Cubic_DG (*B*_i_,t_2_)s at t_2_.

Step 2.2. Generate *Cubic_DG* (*B*_i_,t_2_)s. The *Cubic_DG* (*B*_i_,t_2_)s at t_2_ are generated by synthesizing the *Cubic_DG* (*B_i_*,t_1_)s at t_1_ and the *Slice_DG* (*B_i_*,t_2_)s at t_2_; we then obtain the *Cubic_DG* (*B*_i_,t_2_)s shown in Figure 7. From *Cubic_DG* (*B*_i_,t_2_)s, we can see that *X*_3_ and *X*_5_ did not change, but the state of *X*_6_ changed from normal to abnormal.

Step 2.3. At time t_2_, due to the emergence of the abnormal evidence *X*_6,1_, *Cubic_DB (B_i_*,t_2_)s are regenerated, so the posterior probability of each hypothesis needs to be recalculated under the evidence *E* (t_2_) based on *Cubic_DB* (*B_i_*,t_2_)s. For *H*_1,1_ in *Cubic_DB* (*B*_1_,t_2_):

(18) $$E\left(t_{2}\right)=X_{3,0}X_{5,1}X_{6,1}=F_{3,0;1,1}F_{5,1;1,1}F_{6,1;1,1}B_{1,1}$$

(19) $$\varsigma \left(B_{1},t_{2}\right)=\mathrm{Pr}\left\{E\left(t_{2}\right)\right\}=\mathrm{Pr}\left\{F_{3,0;1,1}F_{5,1;1,1}F_{6,1;1,1}B_{1,1}\right\}=0.004$$

(20) $$H_{1,1}E\left(t_{2}\right)=B_{1,1}X_{3,0}X_{5,1}X_{6,1}=F_{3,0;1,1}F_{5,1;1,1}F_{6,1;1,1}B_{1,1}$$

(21) $$\mathrm{Pr}\left\{H_{1,1}E\left(t_{2}\right)\right\}=\mathrm{Pr}\left\{B_{1,1}X_{3,0}X_{5,1}X_{6,1}\right\}=0.004$$

For *H*_2,1_ and *H*_2,2_ in *Cubic_DB* (*B*_2_,t_2_):

(22) $$E\left(t_{2}\right)=X_{5,1}X_{6,1}=F_{5,1;2,1}F_{6,1;2,1}B_{2,1}+F_{5,1;2,2}F_{6,1;2,2}B_{2,2}$$

(23) $$\varsigma \left(B_{2},t_{2}\right)=\mathrm{Pr}\left\{E\left(t_{2}\right)\right\}=\mathrm{Pr}\left\{F_{5,1;2,1}F_{6,1;2,1}B_{2,1}+F_{5,1;2,2}F_{6,1;2,2}B_{2,2}\right\}=0.16$$

(24) $$H_{2,1}E\left(t_{2}\right)=B_{2,1}X_{5,1}X_{6,1}=F_{5,1;2,1}F_{6,1;2,1}B_{2,1}$$

(25) $$\mathrm{Pr}\left\{H_{2,1}E\left(t_{2}\right)\right\}=0.025$$

(26) $$\mathrm{Pr}\left\{H_{2,2}E\left(t_{2}\right)\right\}=0.135$$

Calculate the posterior probability of each hypothesis according to Equation (3), and we get:

(27) $$\mathrm{Pr}\left\{H_{1,1}\left(t_{2}\right)\right\}=h_{1,1}^{s}\left(t_{2}\right)=\xi_{1}\left(B_{1},t_{2}\right)\frac{\mathrm{Pr}\left\{H_{1,1}E\left(t_{2}\right)\right\}}{\mathrm{Pr}\left\{E\left(t_{2}\right)\right\}}=0.024$$

(28) $$\mathrm{Pr}\left\{H_{2,1}\left(t_{2}\right)\right\}=h_{2,1}^{s}\left(t_{2}\right)=\xi_{1}\left(B_{2},t_{2}\right)\frac{\mathrm{Pr}\left\{H_{2,1}E\left(t_{2}\right)\right\}}{\mathrm{Pr}\left\{E\left(t_{2}\right)\right\}}=0.152$$

(29) $$\mathrm{Pr}\left\{H_{2,2}\left(t_{2}\right)\right\}=h_{2,2}^{s}\left(t_{2}\right)=\xi_{2}\left(B_{2},t_{2}\right)\frac{\mathrm{Pr}\left\{H_{2,2}E\left(t_{2}\right)\right\}}{\mathrm{Pr}\left\{E\left(t_{2}\right)\right\}}=0.823$$

The sorted result is $h_{2,2}^{s}\left(t_{1}\right)>h_{2,1}^{s}\left(t_{1}\right)>h_{1,1}^{s}\left(t_{1}\right)$. With the emergence of new evidence at time t_2_, the ranking probability of each hypothesis changes. From the ranking results, it can be seen that *H*_2,2_ is most likely to cause the abnormal evidence, and the probability of *H*_1,1_ and *H*_2,1_ decreases.

At time t_3_, suppose the received new abnormal evidence is *E* = *X*_4,1_ *X*_7,1_. Then, we get *E* (t_3_) = *X*_3,0_*X*_5,1_*X*_6,1_*X*_4,1_*X*_7,1_.

**Figure 8:**
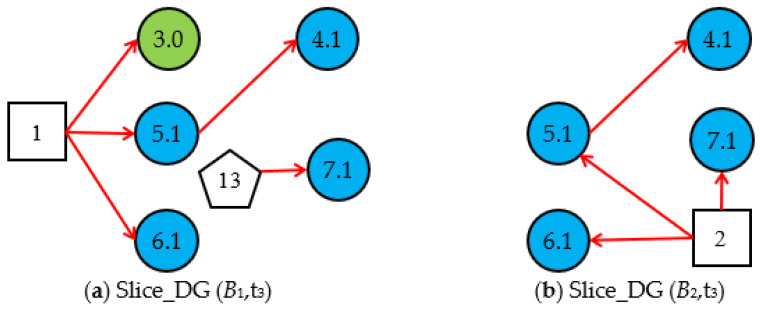
Slice_DG (*B_i_*,t_3_) at t_3_.

Step 3.1. Simplify the DUCG (*B_i_*)s under *E* (t_3_), then we obtain the two *Slice_DG* (*B_i_*,t_3_)s shown in Figure 8. In *Slice_DG* (*B*_1_,t_3_), the abnormal variable *X*_7,1_ is isolated evidence. *H*_1,1_ cannot explain the occurrence of *X*_7,1_, so *Slice_DG* (*B*_1_,t_3_) is regarded as an invalided *Slice_DG* (*B_i_*,t_3_) and deleted. Only *Slice_DG* (*B*_2_,t_3_) is valid.

**Figure 9:**
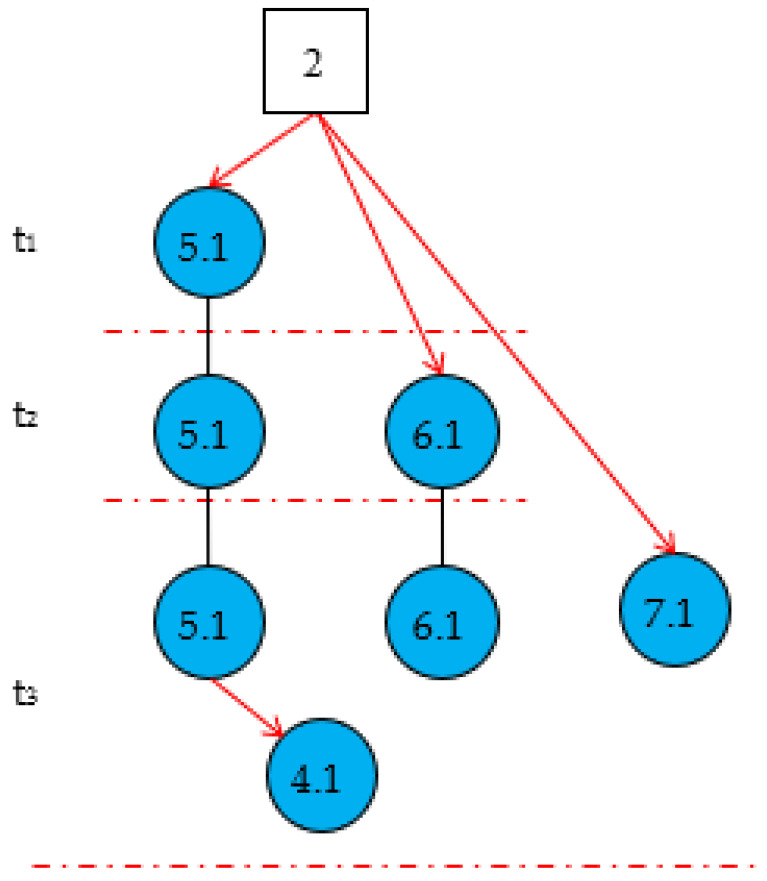
Cubic_DB (*B*_2_,t_3_) at t_3_.

Step 3.2. According to *Cubic_DG* (*B*_2_,t_2_) at t_2_ and *Slice_DG* (*B*_2_,t_3_), generate the *Cubic_DG* (*B*_2_,t_3_) at t_3_, as shown in Figure 9.

Step 3.3. Logical expand *E* (*t*_3_), *H*_2,1_ *E* (*t*_3_), and *H*_2,2_ *E* (*t*_3_) based on *Cubic_DB* (*B*_2_,t_3_) and calculate the joint probability of Pr {*E* (*t*_3_)}, Pr {*H*_2,1_ *E* (*t*_3_)}, Pr {*H*_2,2_ *E* (*t*_3_)} shown in Equations (30)–(35):

(30) $$E\left(t_{3}\right)=F_{4,1;5,1}F_{5,1;2,1}F_{6,1;2,1}F_{7,1;2,1}B_{2,1}+F_{4,1;5,1}F_{5,1;2,2}F_{6,1;2,2}F_{7,1;2,2}B_{2,2}$$

(31) $$\mathrm{Pr}\left\{E\left(t_{3}\right)\right\}=\mathrm{Pr}\left\{F_{4,1;5,1}F_{5,1;2,1}F_{6,1;2,1}F_{7,1;2,1}B_{2,1}+F_{4,1;5,1}F_{5,1;2,2}F_{6,1;2,2}F_{7,1;2,2}B_{2,2}\right\}=0.08085$$

(32) $$H_{2,1}E\left(t_{3}\right)=X_{4,1}X_{5,1}X_{6,1}X_{7,1}B_{1,1}=F_{4,1;5,1}F_{5,1;2,1}F_{6,1;2,1}F_{7,1;2,1}B_{2,1}$$

(33) $$\mathrm{Pr}\left\{H_{2,1}E\left(t_{3}\right)\right\}=\mathrm{Pr}\left\{F_{4,1;5,1}F_{5,1;2,1}F_{6,1;2,1}F_{7,1;2,1}B_{2,1}\right\}=0.00525$$

(34) $$H_{2,2}E\left(t_{3}\right)=X_{4,1}X_{5,1}X_{6,1}X_{7,1}B_{2,2}=F_{4,1;5,1}F_{5,1;2,2}F_{6,1;2,2}F_{7,1;2,2}B_{2,2}$$

(35) $$\mathrm{Pr}\left\{H_{2,2}E\left(t_{3}\right)\right\}=\mathrm{Pr}\left\{F_{4,1;5,1}F_{5,1;2,2}F_{6,1;2,2}F_{7,1;2,2}B_{2,2}\right\}=0.0756$$

Because there is only one *Slice_DG* at *t*_3_, $\xi_{2}\left(B_{2},t_{3}\right)=1$, and according to Equation (3), we get the inference results of *H*_2,1_ and *H*_2,2_ shown in Equations (36) and (37):

(36) $$\mathrm{Pr}\left\{H_{2,1}\left(t_{3}\right)\right\}=\frac{\mathrm{Pr}\left\{H_{2,1}E\left(t_{3}\right)\right\}}{\mathrm{Pr}\left\{E\left(t_{3}\right)\right\}}=0.065$$

(37) $$\mathrm{Pr}\left\{H_{2,2}\left(t_{3}\right)\right\}=\frac{\mathrm{Pr}\left\{H_{2,2}E\left(t_{3}\right)\right\}}{\mathrm{Pr}\left\{E\left(t_{3}\right)\right\}}=0.935$$

The sorted result is $h_{2,2}^{s}\left(t_{1}\right)>h_{2,1}^{s}\left(t_{1}\right)$. Since *H*_1,1_ cannot explain the occurrence of *X*_7,1_ at time t_3_, it is considered an invalid hypothesis and excluded. Both *H*_2,2_ and *H*_2,1_ can explain all abnormal evidence. The probability of *H*_2,2_ causing the abnormal evidence at t_3_ is 0.935, while the probability of *H*_2,1_ causing abnormal evidence is 0.065. According to the results, we can preliminarily judge that the abnormal evidence may be caused by *H*_2,2_.

This was the reasoning process of the cubic DUCG, and the inference was based on a time series. The algorithm reconstructed the current cubic DUCG based on the evidence received at the current moment and the cubic DUCG at the last moment, showing the causal propagation process based on the time series. The DUCG simplification could simplify the complex original DUCG into a set of simple *Slice_DG* (*B_i_*,*t_m_*) according to the evidence *E* (*t*_m_) at *t*_m_. *Slice_DG* (*B_i_*,*t_m_*) described the relationship between the evidence *E* (*t*_m_) and hypothesis *B_i_*. The DUCG simplification could remove the impossible causalities and irrelevant variables on the condition of the evidence *E* (*t*_m_). Meanwhile, the computation scale was reduced exponentially without losing accuracy. The *Cubic_DG* (*B_i_*,*t_m_*) reflected how evidence changed over time slices, and was used as the graphical explanation of the hypothesis *B_i_* and to enhance the interpretability of the inference results. The logical expansion of expression then expressed the logical relationship between the evidence and hypothesis, which is the premise of probability calculation. The reasoning calculation calculated the conditional probability of each hypothesis under the current evidence according to the causal effect between variables.

## 3. System Design

**Figure 10:**
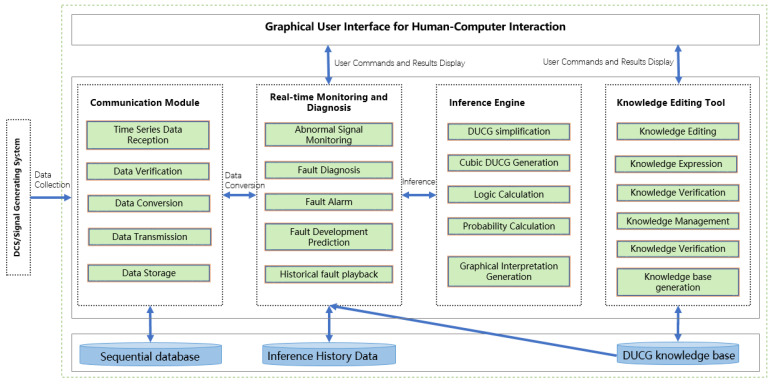
System block diagram of the cubic-DUCG-based industrial fault diagnostic system.

According to the reasoning mode of cubic DUCG and the characteristics of industrial diagnostic systems, the cubic-DUCG-based industrial fault diagnostic system was divided into four parts: the communication module, real-time monitoring and diagnosis module, inference engine, and knowledge-editing tool. The summary of each functional module is shown in Figure 10.

**Figure 11:**
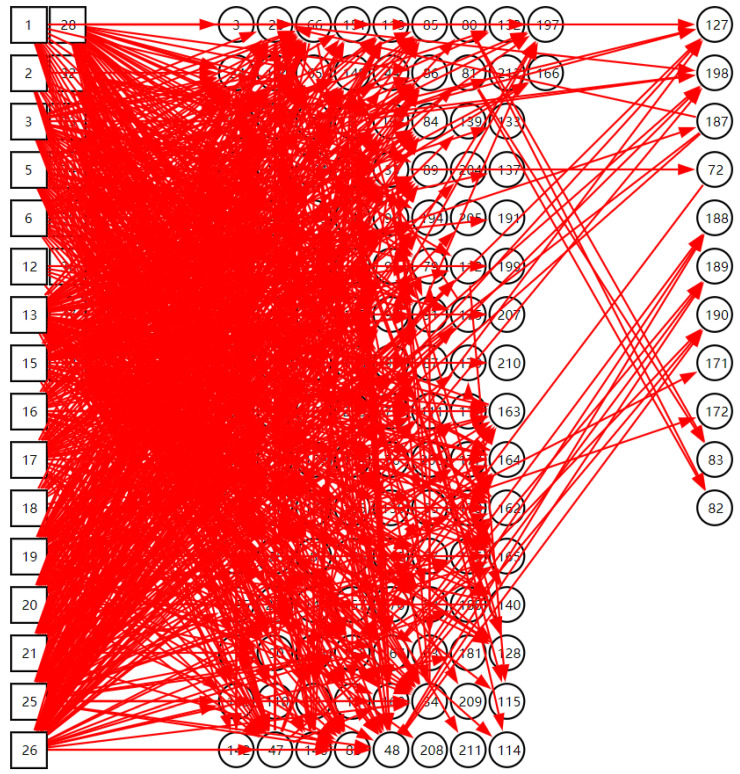
The DUCG knowledge base for the secondary circuit of No. 1 at the Ningde nuclear power plant.

The knowledge-editing tool was used by domain experts to design the DUCG knowledge base. The DUCG knowledge base can be built in a modular way, and a whole DUCG can be divided into several sub-DUCGs. Generally, each sub-DUCG contains one fault, and represents causal relations between the fault and its related monitoring signals. This modular method of knowledge-base construction could reduce the difficulty in constructing a large complex knowledge base. Figure 11 shows a complete DUCG knowledge base. It was used for the fault diagnosis of the secondary circuit of No. 1 at the Ningde nuclear power plant. This DUCG knowledge base is reconstructed from the knowledge base in paper [35]. It contains 24 *B*-type variables that represent 24 different root faults in the secondary circuit of the nuclear power water reactor; the detail of the faults are shown in Appendix B. 141 *X*-type variables were used to describe the intermediate process or results arising from a root fault; a total of 1192 *F*-type variables (the direct red line) were used to describe the causal relations among variables.

The communication module was used for signal processing. The communication module received the monitoring data from the industrial system. It transformed the data to conform to the data format requirements of DUCG according to the mapping relationship between the measure points and variables, then transmitted the data to the real-time monitoring and diagnosis module.

The inference engine was the core module of the fault diagnostic system. It could generate cubic DUCG and engage in continuous causal reasoning based on the abnormal evidence. Its diagnostic results were presented in probabilistic form, and the generated cubic DUCG was used to explain the results. The inference engine was an independent service. Its data resulted from the real-time monitoring module, and its inference results were sent back to the real-time monitoring module for user decision making.

The real-time monitoring and diagnosis module was the control and human–computer interaction center. In this module, users could choose the DUCG knowledge base based on monitoring requirements, and then the instruction was sent to the communication module to receive and process signals associated with the current DUCG knowledge base. The monitoring module displayed and monitored signals in real-time.

The four functional modules of the system completed the functions of building a knowledge base, receiving and processing data, diagnosing faults, and displaying results. Through this system, users could translate knowledge and experience into diagnostic models. Furthermore, the model was used for real-time fault diagnosis. The system was a web application, the web client of the system was implemented with jquery+html, and the server–client was implemented using Java; the framework adopted by the system was the spring boot. The system is developed based on cloud native technology. Compared with the DUCG system based on traditional web technology [35]. The system has good scalability and can dynamically increase computing power according to task requirements.

## 4. Experiment

In order to validate the feasibility and diagnostic accuracy of the system, an experiment was done based on the secondary circuit of No. 1 at the Ningde nuclear power plant. The DUCG knowledge base is shown in Figure 11. The system was deployed on the computer cluster. The inference engine was deployed on one machine of the cluster with an AMD Ryzen 7 5700G CPU at 4.45 GHz, an 8-core processor, and 128 GB of RAM. The test data were collected from the simulator of the secondary circuit of No. 1 at the Ningde nuclear power plant. A total of 24 fault cases were used to test the system. Each fault case contained several time slices, and each time slice contained 141 signal data. The data types of the signals included switching value, continuous data, and discrete data. These signal data corresponded to 141 variables in the model one by one. During the system test, the simulator sent a group of signal data to the system every other second, which was recorded as a time slice. An example of condensate extraction pump failure was used to demonstrate the diagnostic process of the system.

**Figure 12:**
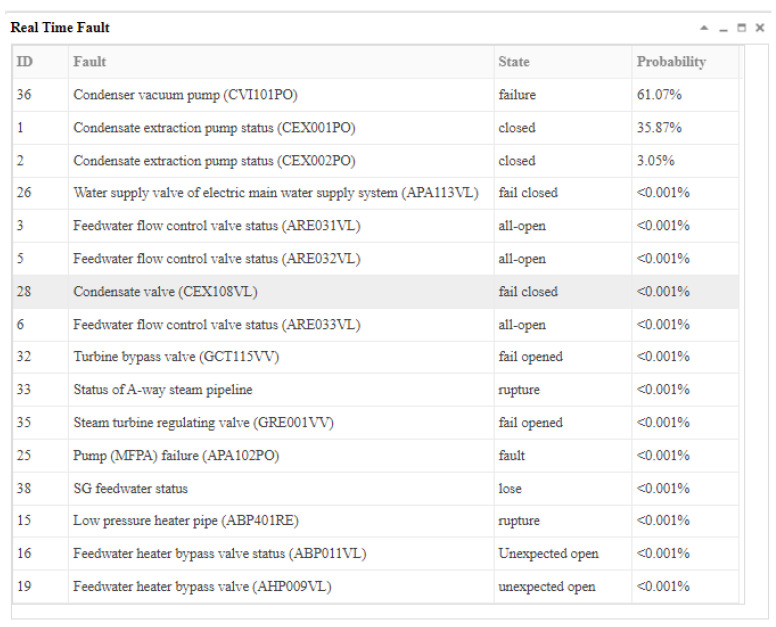
The inference results at t_1_ according to the abnormal evidence *E* = *X*_71,2_.

**Figure 13:**
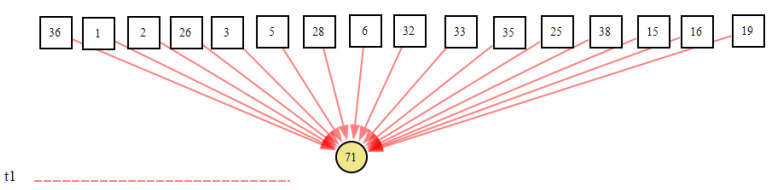
The graphical interpretation of the inference hypothesis and abnormal evidence at *t*_1_.

The fault “condensate extraction pump fault (CEX001PO)” was inserted at the 13th second after the simulator operated stably, and the opening of the pump CEX001PO gradually decreased; then, when the communication module received the real-time data at the 14th second, one of the variables was in its abnormal state (the intake pressure of ABP401RE was low (ABP004MP), *X*_71,2_). Because this was the first time the system received the abnormal evidence, this time was marked as t_1_ and the system started the inference. The inference results and graphical explanation at t_1_ are shown in Figure 12 and Figure 13.

Figure 13 shows that because multiple fault sources could cause *X*_71,2_ to occur, the fault source cannot be diagnosed at *t*_1_, but the scope of the fault and the probability of each fault could be preliminarily inferred. Sixteen faults could cause *X*_71,2_ to occur, and the top three faults in the result list were more likely to cause *X*_71,2_ to occur.

**Figure 14:**
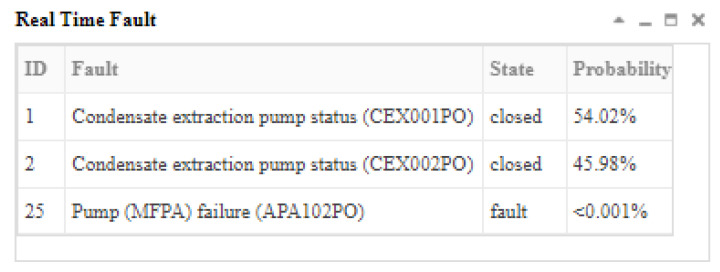
The inference results at *t*_2_ according to the abnormal evidence *E* = *X*_71,2_ *X*_195,1_.

**Figure 15:**
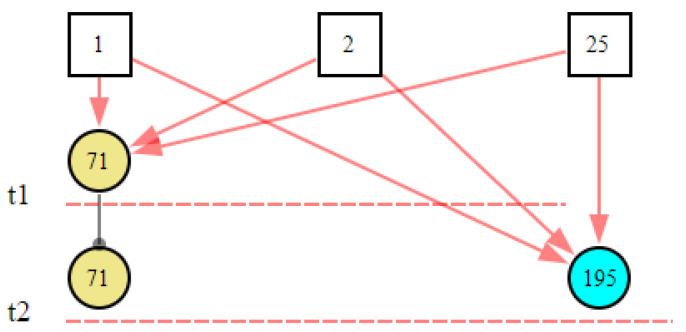
The graphical interpretation of the inference hypothesis and abnormal evidence at *t*_2_.

At the 15th second, the system did not receive the new abnormal evidence, so it did not perform the reasoning calculation. At the 16th second, the communication module received the new abnormal signal (condensate extraction pump (CEX003PO) failure, *X*_195,1_). This time was marked as *t*_2_. The inference results and graphical explanation are shown in Figure 14 and Figure 15. Comparing the inference results in Figure 12 and Figure 14, we can see that hypothesis space was further reduced. The hypotheses in the first inference that could not explain the new evidence were excluded. Only the hypotheses that could explain all abnormal evidence were valid. According to the results, we could infer that the abnormal signals were possibly caused by the condensate extraction pump status (CEX001PO) (*B*_1,1_) or the condensate extraction pump status (CEX002PO) (*B*_2,1_), and *B*_1,1_ was more likely.

**Figure 16:**
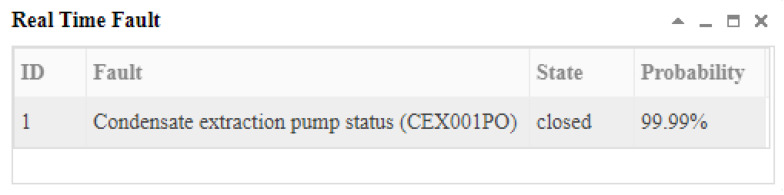
The third inference results when more abnormal evidence was received.

**Figure 17:**
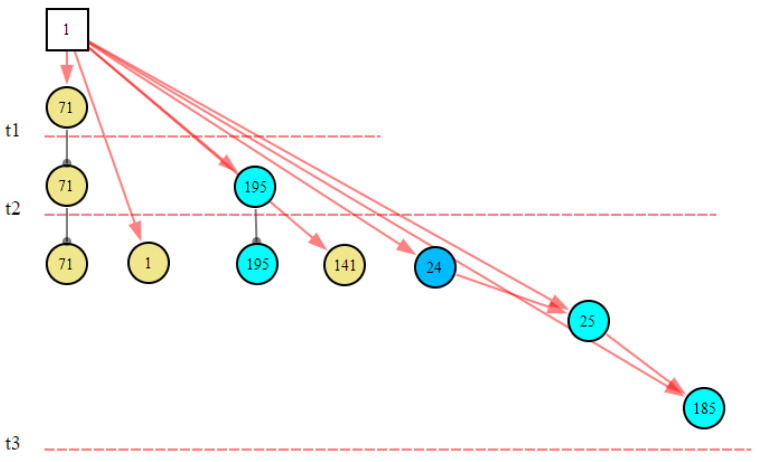
The graphical interpretation of the inference hypothesis and abnormal evidence at *t_3_*.

At the 17th second, the communication system received more abnormal signals. This time was marked as time *t_3_*. The third inference results and graphical interpretation are shown in Figure 16 and Figure 17. Because only *B*_1,1_ could explain all of the known abnormal evidence, according to the current evidence, *B*_1,1_ was diagnosed.

This example showed the inference process of the cubic DUCG. It performed inference calculations based on the time series. This fault diagnosis included three moments, and the moment that first showed the abnormal evidence was marked as *t*_1_. At *t_1_*, there was less abnormal evidence, so the specific diagnosis result could not be determined, but the range of possible failures could be roughly determined. At *t_2_*, new abnormal evidence was added to the diagnosis, which further narrowed the scope of the fault diagnosis. At *t_3_*, new evidence increased, and the fault was uniquely determined. In the next few moments, new evidence continued to be received. However, since there was only one diagnostic fault left and the fault could explain all abnormal evidence, the diagnosis result did not change, and the diagnosis was completed. At each moment, the system dynamically generated a new cubic DUCG for the diagnosis combined with the cubic DUCG obtained from the diagnosis at the last moment and the new evidence, giving the reasoning diagnosis results. The graphical interpretations could demonstrate the development and evolution process of the fault with time. It was convenient for the operator to understand the development of the fault for troubleshooting.

Table 2 shows the test results of the 24 fault cases. The “Fault” is the fault code in the DUCG knowledge base. “Rank First” indicates the first moment when the fault was ranked first in the diagnostic results. “Confirmed Diagnosis” indicates the moment when the fault was confirmed. “Time Consumption” indicates the total reasoning time for diagnosing the fault. “Average Time” represents the average time of each diagnosis. We can see that the 24 fault cases were all correctly diagnosed from the results. This proved that the fault-diagnosis model of the secondary circuit system constructed in this study was accurate. It also proved that using the cubic DUCG to construct a complex diagnostic model was feasible. Among the 24 fault cases, 17 of them could be diagnosed at time *t*1. The remaining seven faults needed multiple time slices to be diagnosed, but they could all be sorted to the first place in the list of diagnostic faults within four diagnostic time slices, which proved that the system could quickly locate the root faults in a short time. In terms of inference calculation time, although the inference calculation time increased with an increase in the diagnosis time slice, the average time (from the start of the diagnosis to the fault being confirmed) for each diagnosis was within 400 ms. Compared with the DUCG-based diagnostic system proposed by Zhao [35], the diagnostic efficiency of the system in this study is more efficient. This showed that the system could complete a real-time and efficient reasoning diagnosis and meet the real-time task requirements of industrial diagnosis.

## 5. Conclusions

With the rapid development of science and technology in industrial systems, such as space systems and nuclear power plants, those systems have shown the characteristics of increasingly complex structures, increasing scales, and increasingly rich functions, as well as gradual improvements in the levels of integration and automation. Once an accident occurs in a complex industrial system, it may cause casualties, damage to equipment and facilities, damage to the ecological environment, and loss of economic property. These systems have high security, and the fault data used for model training is insufficient. In addition, these systems are usually customized according to different task requirements, so the fault data between different systems lacks universality. Therefore, it is not feasible to use big data to train models for fault diagnosis. However, experts accumulate a great deal of experience and knowledge when designing and constructing such systems. At the same time, the fault data of other similar systems can also provide a reference for the fault analysis of the system. Therefore, some diagnostic methods based on expert knowledge are more suitable for the fault diagnosis of these systems. Traditional fault-diagnosis methods, such as the rule-based method, fault tree, and event tree, have the disadvantages of a low reasoning efficiency, an inability to deal with the logical cycle or uncertain causalities, and difficulty in managing the growing knowledge.

This study proposed a fault-diagnosis method for a unique industrial system based on the cubic DUCG. The model was built based on expert knowledge, experience, and statistical data, and it described the causal mechanism of faults and abnormal signals. The system could carry out a continuous fault diagnosis according to the time sequence, display the results in the form of probability, and graphically represent the propagation of faults over time. The modular construction method of the cubic DUCG knowledge base reduced the modeling difficulty for large and complex knowledge bases and facilitated knowledge management and maintenance. The cubic DUCG could express logical cycles and uncertain causal relationships to express expert knowledge accurately. The reasoning process of the cubic DUCG included model simplification, logical calculation, and probability calculation. This inference method reduced the computational complexity of inference without losing the accuracy of the results. It solved the problem of the high computational complexity of large and complex knowledge base reasoning. These characteristics of the cubic DUCG made it more suitable for industrial system modeling and fault diagnosis based on expert knowledge.

In order to verify the feasibility and effectiveness of the system, we cooperated with nuclear experts to build the fault diagnostic model of the secondary circuit system of No. 1 at the Ningde nuclear power plant. The model’s 24 root faults represented the operation status of 24 leading components of the secondary circuit system, such as the steam turbines and electric generators. A total of 141 variables represented the abnormal signals that root faults may have caused. The data types of variables included switching value, discrete type, and continuous type. Experts determined the values of the causal strength between variables based on experience or statistical data. The variables in the model could be mapped one-to-one with the detection points in the secondary circuit system. They could reflect the operation state of the secondary circuit system. The secondary system simulator generated 24 groups of fault cases to test the model. Each case tested 1 root fault, and 24 faults were correctly diagnosed. The test results showed that using the cubic DUCG for fault diagnosis in unique industrial systems was feasible. At the same time, it should be pointed out that the diagnostic accuracy of the diagnosis system based on expert knowledge depended on the accuracy of the model. Therefore, multiexpert joint modeling and third-party auditing of the model is one of the methods to ensure the model’s accuracy. Since the current verification data were only provided by the nuclear power plant simulator, the verification model was also limited to 24 faults in the secondary circuit system of the nuclear power plant. Therefore, the verification of the system with only one application scenario was not complete and systematic. In a following work, we will continue to extend the model to test it using more data. In addition, we will verify the performance of the system in more application scenarios.

## Figures and Tables

**Table 1:** The physical meanings of variables in the DUCG.

Type	Shape	Description
*B_i_*	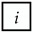	The *B*-type variable is the root-cause variable used to represent the root cause/fault that causes other variables to occur.
*X_i_*	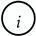	The *X*-type variable is the consequence or process variable used to represent the result caused by the root-cause variable, and can also be used as the cause of other variables.
*G_i_*	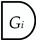	The *G*-type variable is the logic-gate variable. It is used to describe the logical relation combination of parent variables.
*D_i_*	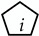	The *D*-type variable is the unknown cause or default-cause variable. When the cause of a variable’s occurrence is unknown, then the *D*-type variable is used to represent the root cause that causes it to occur.
*F_i;j_*	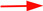	The *F*-type variable is the weighted functional-event variable. It is used to represent and quantify the causalities between parent variables and child variables.

**Table 2:** The diagnostic results of the 24 faults of the secondary circuit of No. 1 at Ningde nuclear power plant.

Fault	Rank First	Confirmed Diagnosis	Time Consumption	Average Time
*B*_1,1_	t_2_	t_3_	145 ms	48 ms
*B*_2,1_	t_1_	t_1_	19 ms	19 ms
*B*_3,2_	t_1_	t_1_	18 ms	18 ms
*B*_5,2_	t_1_	t_1_	19 ms	19 ms
*B*_6,2_	t_1_	t_1_	18 ms	18 ms
*B*_13,1_	t_1_	t_1_	313 ms	313 ms
*B*_15,1_	t_1_	t_1_	26 ms	26 ms
*B*_16,1_	t_3_	t_8_	437 ms	55 ms
*B*_17,1_	t_1_	t_1_	21 ms	21 ms
*B*_18,1_	t_1_	t_1_	20 ms	20 ms
*B*_19,1_	t_3_	t_8_	433 ms	55 ms
*B*_20,1_	t_1_	t_1_	274 ms	274 ms
*B*_21,1_	t_1_	t_1_	276 ms	276 ms
*B*_25,1_	t_1_	t_1_	81 ms	81 ms
*B*_26,1_	t_2_	t_2_	188 ms	94 ms
*B*_28,1_	t_1_	t_1_	23 ms	23 ms
*B*_32,1_	t_1_	t_1_	142 ms	142 ms
*B*_33,1_	t_1_	t_1_	230 ms	230 ms
*B*_34,1_	t_4_	t_4_	496 ms	124 ms
*B*_35,1_	t_1_	t_12_	1874 ms	157 ms
*B*_35,2_	t_1_	t_1_	31 ms	31 ms
*B*_36,1_	t_1_	t_1_	18 ms	18 ms
*B*_37,1_	t_1_	t_1_	21 ms	21 ms
*B*_38,1_	t_2_	t_2_	398 ms	199 ms

## Data Availability

Not applicable.

## Appendix A. The Parameters of the Example DUCG Knowledge Base

(A1)
b1=−0.2T, b2=−0.10.3T, b8=−0.3T, a3;1=−−−0.5,a4;4=−−−0.2, a5;1=−−−0.1, a4;5=−−−0.7, a6;1=−−−0.4,a6;2=−−−−0.50.9, a5;2=−−−−0.50.5, a7;2=−−−−0.30.8,a3;8=−−−0.1, a9;8=−−−0.9, LGS11=0Remnant1X3,1X9,1,a10;11=−−−0.9, ri=1.

## Appendix B. Description of 24 Faults in the Knowledge Base, Including Fault Name, State Description, and Its Number in DUCG

**Table A1:** The root faults in the cubic DUCG of the secondary circuit of No. 1 at Ningde nuclear power plant.

ID	Fault Description	State	State Description
*B*_1_	Condensate extraction pump status (CEX001PO)	0	Working order
		1	Closed
*B*_2_	Condensate extraction pump status (CEX002PO)	0	Working order
		1	Closed
*B*_3_	Feedwater flow control valve status (ARE031VL)	0	Normal
		1	All closed
		2	All opened
*B*_5_	Feedwater flow control valve status (ARE032VL)	0	Normal
		1	All closed
		2	All opened
*B*_6_	Feedwater flow control valve status (ARE033VL)	0	Normal
		1	All closed
		2	All opened
*B*_12_	Water supply pipeline of route A	0	Normal
		1	Leaked
*B*_13_	Turbine operation status	0	Working order
		1	Tripping state
*B*_15_	Low pressure heater pipe (ABP401RE)	0	Normal
		1	Rupture
*B*_16_	Feedwater heater bypass valve status (ABP011VL)	0	Normal
		1	Accidental opening
*B*_17_	Main steam header status	0	Normal
		1	Leaked
*B*_18_	Steam generator heat transfer tube	0	Normal
		1	Leaked
*B*_19_	Feedwater heater bypass valve (AHP009VL)	0	Normal
		1	Accidental opening
*B*_20_	Water supply pipeline of route B	0	Normal
		1	Leaked
*B*_21_	Water supply pipeline of route C	0	Normal
		1	Leaked
*B*_25_	Pump (MFPA) failure (APA102PO)	0	Normal
		1	Fault condition
*B*_26_	Water supply valve of electric main water supply system (APA113VL)	0	Normal
		1	Accidental shutdown
*B*_28_	Water supply valve of electric main water supply system (APA113VL)	0	Normal
		1	Accidental shutdown
*B*_32_	Turbine bypass valve (GCT115VV)	0	Normal
		1	Accidental opening
*B*_33_	Status of A-way steam pipeline	0	Normal
		1	Rupture
*B*_34_	Turbine bypass valve (GCT131VV)	0	Normal
		1	Accidental opening
*B*_35_	Steam turbine regulating valve (GRE001VV)	0	Normal
		1	Accidental all closed
		2	Accidental all opened
*B*_36_	Condenser vacuum pump (CVI101PO)	0	Normal
		1	Accidental failure
*B*_37_	Status of three main steam isolation valves (VVP001/002/003VV)	0	Normal
		1	All closed
*B*_38_	SG feedwater status	0	Normal
		1	Loss
		2	Moderate
